# Reply to the letter by Dr Karlsson ‘Rationale behind the European Thyroid Association 2024 Guideline to treat the Allan–Herndon–Dudley syndrome with tiratricol?’

**DOI:** 10.1530/ETJ-25-0256

**Published:** 2025-10-24

**Authors:** Luca Persani, Patrice Rodien, Carla Moran, W Edward Visser, Stefan Groeneweg, Robin Peeters, Samuel Refetoff, Mark Gurnell, Paolo Beck-Peccoz, Krishna Chatterjee

**Affiliations:** ^1^ Department of Endocrine and Metabolic Diseases, IRCCS Istituto Auxologico Italiano, Milano, Italy; ^2^ Department of Medical Biotechnology and Translational Medicine, University of Milan, Milano, Italy; ^3^ Service d’Endocrinologie-Diabétologie-Nutrition, Centre de référence des maladies rares de la Thyroïde et des récepteurs hormonaux, CHU d’Angers, Angers, France; ^4^ Institute of Metabolic Science, University of Cambridge, Cambridge, UK; ^5^ Endocrine Section, Beacon Hospital, Dublin, Ireland; ^6^ School of Medicine, University College Dublin, Dublin, Ireland; ^7^ Endocrinology Department, St Vincent’s University Hospital, Dublin, Ireland; ^8^ Department of Internal Medicine and Rotterdam Thyroid Center, Erasmus University Medical Center, Rotterdam, The Netherlands; ^9^ Departments of Medicine and Paediatrics and Committee on Genetics, The University of Chicago, Chicago, Illinois, USA

**Keywords:** clinical practice guideline, deiodinase, diagnosis and management, impaired sensitivity to thyroid hormones, resistance to thyroid hormone, selenoprotein, thyroid hormone receptor, thyroid hormone transporter

Dear Editor,

We thank Dr Karlsson for his letter (1), which provides us with an opportunity to further clarify the basis on which we recommend considering tiratricol treatment, if available.

Analogous to the 2022 European Thyroid Association Guideline for the management of paediatric Graves’ disease (2), Dr Karlsson suggests considering total thyroidectomy followed by levothyroxine supplementation in patients with MCT8 deficiency. In response, we wish to point out that the pathophysiology of Graves’ disease and MCT8 deficiency are completely different. In Graves’ disease, stimulating anti-TSH-receptor antibodies drive overproduction of hormones from the thyroid gland in an individual whose tissues are equally sensitive to thyroid hormones. In contrast, in MCT8 deficiency, the cause of raised circulating thyroid hormones is more complex, with tissues exhibiting different sensitivity to thyroid hormone action (3).

To the best of our knowledge, a single report has recorded the effects of thyroidectomy (for suspected thyroid cancer) followed by levothyroxine treatment in an individual with MCT8 deficiency (4). The authors showed that, following thyroidectomy, replacement with levothyroxine in a dosage sufficient to normalize TSH concentrations was associated with persistently low (F)T4 together with raised serum T3 concentrations. This thyroid hormone profile is not different from untreated patients and is known to be associated with adverse sequelae due to the thyrotoxic status of peripheral tissues. This biochemical pattern also accords with knowledge that increased type 1 deiodinase (DIO1) activity is a major contributor to abnormal thyroid function seen in MCT8 deficiency (5). Furthermore, although treating patients with MCT8 deficiency with a combination of propylthiouracil (PTU) to inhibit DIO1 activity and levothyroxine improves their thyroid function tests, no statistically significant changes in metabolic and anthropometric outcomes were seen (6, 7). As it can cause unpredictable hepatotoxicity, which can be fatal, the 2022 European Thyroid Association Guideline for the management of paediatric Graves’ disease recommends against the use of PTU in this context (2). For this reason, in the current guideline, we have suggested that PTU and thyroxine can be used in MCT8 deficiency, but with great caution.

Taking the pathophysiological basis and current knowledge of different therapies in MCT8 deficiency into account, we feel that there is no evidence to advocate a thyroidectomy followed by levothyroxine replacement in patients with this disorder.

Recommendations in guidelines should be based on the best evidence available at the time they are formulated. For the record, we wish to restate that our recommendations in MCT8 deficiency were based on important trial and outcome data (8, 9), which may also have informed EMA approval of tiratricol. Clearly, should new, better treatments become available in the future, our recommendations could change. We strongly encourage endeavours to develop and trial new therapies for MCT8 deficiency, aiming to fulfil the unmet needs of patients with this debilitating disease.

## Declaration of interest

The task force had no commercial support, and LP, PR, SR, MG, PBP, and KC have no conflicts of interest to declare. CM has consulted for Egetis Therapeutics. The Erasmus Medical Centre (Rotterdam, Netherlands), which employs RP, WEV, and SG receives royalties from Egetis Therapeutics (a manufacturer of TRIAC), dependent on commercialization. None of the authors will benefit personally from any royalties.

## Funding

MG and KC are supported by the NIHR Cambridge Biomedical Research Centrehttps://doi.org/10.13039/501100018956 (NIHR203312). KC is supported by a Wellcome Trusthttps://doi.org/10.13039/100010269 Investigator Award (210755/Z/18/Z) and Medical Research Councilhttps://doi.org/10.13039/501100000265 funding (MRC_MC_UU_00014/40). SR is supported by a grant (DK15070) from the National Institutes of Healthhttps://doi.org/10.13039/100000002, USA. LP is supported by funding from the Italian Ministry of Health, Rome, Italy (Ricerca Corrente and PNRR-MR1-2022-12375726; RTH2018, 05C821_2018). PR is supported by the plan maladies rares of the French health care ministry. WEV is supported by ZonMwhttps://doi.org/10.13039/501100001826 (Dutch Research Council, VIDI 09150172210071). LP, PR, WEV, SG, and RP are members of the European Reference Network on Rare Endocrine Conditions (Endo-ERN), which is co-funded by the European Union’s 3rd Health Programme (CHAFEA Framework Partnership agreement no. 739527).
